# Angiogenic Imbalance in Early Placental Dysfunction: Pro- and Anti-Angiogenic Factors, Pathophysiology, Screening and Therapeutic Horizons

**DOI:** 10.3390/ijms27208958

**Published:** 2026-10-09

**Authors:** Tamara Nikolic Turnic, Marija Aksentijevic

**Affiliations:** 1 Department of Pharmacy, Faculty of Medical Sciences, University of Kragujevac, Svetozara Markovica 69, 34000 Kragujevac, Serbia; 2 N.A. Semashko Public Health and Healthcare Department, F.F. Erismann Institute of Public Health, I.M. Sechenov First Moscow State Medical University ( Sechenov University), 119048 Moscow, Russia; 3 Center of Excellence for Redox Balance Research in Cardiovascular and Metabolic Disorders, 34000 Kragujevac, Serbia

**Keywords:** angiogenic factors, placental dyfunction, sFlt-1/PlGF ratio, PIGF, VEGF, eEng, TGF-beta, sFlt-1

## Abstract

Placental dysfunction is a major component of several pregnancy complications, particularly preeclampsia and fetal growth restriction (FGR). These disorders could be presented with different phenotypes based on abnormal placentation and inadequate maternal vascular adaptation, which are baseline mechanisms in affected pregnancies. Defective trophoblast invasion and incomplete transformation of the maternal spiral arteries may produce a high-resistance uteroplacental circulation, resulting in placental hypo-perfusion, ischemia–reperfusion injury, oxidative stress and subsequent release of factors that affect maternal endothelial function. The aim of this review is to summarize the current understanding of pro-angiogenic and anti-angiogenic factors in early placental dysfunction, with special emphasis in their biological function and molecular roles, relationship with abnormal placentation and clinical significance during pregnancy. The development of placental dysfunction is generally understood as a multifactorial and multistep process. The literature evidence most often supports the three-step process of placenta dysfunction. The angiogenic imbalance may precede the clinical presentation of preeclampsia and may also be associated with fetal growth restriction. Among currently available biomarkers, the sFlt-1/PlGF ratio has the strongest clinical evidence for risk stratification in women with suspected preeclampsia. Nevertheless, the role of angiogenic factors in the earliest stages of placental dysfunction remains incompletely defined. The major challenge is to determine whether angiogenic abnormalities can identify clinically meaningful placental dysfunction early enough to permit effective intervention. This suggests that pro-angiogenic therapeutics could be a possible option for the treatment of pre-eclampsia, but also a more specific and sensitive predictive and diagnostic tool in modern obstetrics.

## 1. Introduction

The placenta is a highly specialized vascular organ that undergoes profound structural and functional changes during pregnancy. In pregnancy, different physiological changes occur, and a successful pregnancy depends on the balance of an adequate uteroplacental circulation, delivers oxygen and nutrients to the fetus, and removes metabolic waste on the other side. Very important processes are: invasion of trophoblast, remodeling of spiral artery, angiogenesis of the placental vessels and maternal cardiovascular system adaptation [1,2,3].

Placental dysfunction is a major component of several pregnancy complications, particularly preeclampsia and fetal growth restriction (FGR). These disorders could be presented with different phenotypes based on abnormal placentation and inadequate maternal vascular adaptation, which are baseline mechanisms in affected pregnancies. Defective trophoblast invasion and incomplete transformation of the maternal spiral arteries may produce a high-resistance uteroplacental circulation, resulting in placental hypo-perfusion, ischemia–reperfusion injury, oxidative stress and subsequent release of factors that affect maternal endothelial function [4,5,6].

In this complex process of placental dysfunction, the main actors are angiogenic and anti-angiogenic factors which contribute to physiological balance, essential for normal placental vascular development and maternal endothelial homeostasis. Disruption of this balance, particularly an increase in one of them, such as circulating sFlt-1, is accompanied by reduced free PIGF and VEGF activity, together representing the central failure of the biological model of preeclampsia [7,8].

It is very important to detect imbalance of angiogenic factors early and prevent clinical disease. This observation has generated substantial interest in angiogenic biomarkers as tools for identifying women at increased risk of placenta-mediated complications and for distinguishing established disease from other causes of hypertension or nonspecific pregnancy symptoms [7,8,9].

The aim of this review is to summarize the current understanding of pro-angiogenic and anti-angiogenic factors in early placental dysfunction, with special emphasis on their biological function and molecular roles, relationship with abnormal placentation and clinical significance during pregnancy.

### Literature Search Strategy

A comprehensive literature search was conducted across PubMed/MEDLINE, Scopus, and the Cochrane Library databases to identify relevant studies published from database inception up to the 15th of August 2026. The search strategy employed combinations of Medical Subject Headings (MeSH) terms and free-text keywords related to the core themes of the review. The primary search string included: (“placental dysfunction” OR “abnormal placentation”) AND (“angiogenic factors” OR “anti-angiogenic factors” OR “sFlt-1” OR “PlGF” OR “sFlt-1/PlGF ratio”) AND (“preeclampsia” OR “fetal growth restriction” OR “FGR” OR “maternal vascular adaptation”).

The selection criteria focused on peer-reviewed original research articles, prospective cohort studies, clinical trials, and high-impact systematic reviews published primarily in the English language. Spatial and temporal prioritization was given to recent literature (within the last 10 years) focusing on early pregnancy prediction, multi-step pathogenesis (the three-step model), and emerging pro-angiogenic therapeutics. Studies were evaluated and prioritized based on their methodological robustness, sample size, and direct relevance to the molecular mechanisms and clinical utility of angiogenic biomarkers in early gestation.

## 2. Physiologically Processes in Angiogenesis During Pregnancy

The establishment of a low-resistance, high flow uteroplacental circulation is a prerequisite for a healthy pregnancy. This process relies on a coordinated vascular network governed by two sequential phases: vasculogenesis and angiogenesis. During early embryonic development, vasculogenesis initiates the de novo formation of the first primitive capillary networks from pluripotent mesenchymal cells within the chronic villi. Subsequently, angiogenesis takes over, driving proliferation, sprouting, elongation and non-sprouting intussusceptive remodeling of these pre-existing vessels throughout gestation. In vital pregnancy, angiogenesis represents the growth of new blood vessels from existing ones to build the placenta and complex capillary networks required to supply oxygen and nutrients from the mother to the growing fetus. In early gestation, placental angiogenesis begins at the start and continues throughout pregnancy [10,11]. Trophoblasts invade the maternal decidua and contribute to remodeling of the spiral arteries and to placental development. These arteries intensively lose their muscular and vasoconstrictive characteristics and become low-resistance vessels capable of supplying the intervillous space with consequently increased blood flow. At the similar time, placental vessels related to the fetus undergo angiogenesis and vascular remodeling, which are regulated by various growth factors, receptors, extracellular matrix proteins and mechanisms related to oxygen transport [12,13,14].

In the physiologically process of angiogenesis, the main pro-angiogenic mediators are placental growth factor (PIGF), vascular endothelial growth factor (VEGF) and transforming growth factor beta (TGF-beta), which tend to be in balance with anti-angiogenic factors: soluble fms-like tyrosine kinase-1 (sFlt-1) and soluble endoglin (sEng). While the pro-angiogenic factors help build new blood vessels and keep the lining of blood vessels healthy, anti-angiogenic factors slow down blood vessel growth and can block pro-angiogenic signals when released in high amounts [15,16,17].

VEGF and PIGF are the most important pro-angiogenic factors and mediators of the process of angiogenesis. They achieve their effects through VEGF receptors among endothelial and other placental cells. According to the gestational age and cellular compartment, the exact role could vary. Therefore, a physiological angiogenic environment is characterized not simply by high concentrations of pro-angiogenic factors, but by an appropriate balance between signals promoting vascular growth and mechanisms limiting excessive angiogenesis [18].

### 2.1. Vascular Endothelial Growth Factor (VEGF) in Placental Dysfunction

VEGF is a protein that stimulates the growth of new vessels, or the process of angiogenesis. In normal placentation VEGF has many roles in vessel growth, endothelial health and immune tolerance [19,20]. In detail, this protein drives the formation and branching of the placental capillary network, then promotes the survival and growth of cells lining blood vessels and helps establish a healthy environment for the growing placenta. In placental dysfunction, VEGF could be lowered by the anti-angiogenic factors especially in the state of hypoxia. When the poor blood flow causes low oxygen, it can trigger more VEGF production and the receptor blocks disrupt the signal. Also, a low amount of free VEGF harms maternal blood vessels, raising blood pressure and causing organ stress. The low oxygen or hypoxia is the main reason for the synthesis of VEGF, which is a cellular process. Cells make hypoxia-inducible factor (HIF) which turns on the VEGF gene. Then, the cell creates VEGF mRNA, splices it into different forms, translates it into protein and secretes it to build new blood vessels. These four steps are essential in the process of VEGF synthesis, which is strongly dependent on the cells activation and receptor binding in a signal cascade [19,20,21].

The biological role of VEGF is represented in the regulation of endothelial cell proliferation, migration, vascular permeability and cell survival. The main biological role of VEGF is reflected in VEGF signaling which is essential for vascular development and maintenance of endothelial integrity and strongly depends on VEGF interaction with specific receptors (binding proteins) and soluble receptor forms.

Vascular endothelial growth factors receptors (VEGFRs) are cell-surface proteins that bind VEGF ligands to control the growth of blood and lymphatic vessels. There are three known types of receptors: VEGFR-1, VEGFR-2 and VEGFR-3 which regulate blood vessel formation, cell signals and pathological processes. They belong to the tyrosine kinases group of receptors and are placed on cell membrane with parts outside to connect with signals and transport the massages inside the cell. VEGFR-1 binds VEGF-A, VEGF-B and placental growth factor, with the characteristics of strong binding and weak cell activation, usually works as a negative regulator to balance blood vessel growth. VEGF-2 is the main working receptor which binds VEGF-A and triggers most signals for endothelial cell division, migration and survival. VEGF-3 binds VEGF-C and VEGF-D and it is mostly present in lymphatic endothelial cells to guide the growth of the lymphatic system and regulate the process of lymphangiogenesis. Activation of receptors is based on connection of VEGF protein to the outside part of cell, then dimerization and phosphorylation process [19,20,21].

VEGF has many clinical applications. First, VEGF plays a fundamental role in regulating angiogenesis. In clinical practice, VEGF could be used in anti-VEGF therapy, which inhibits blood vessel growth in tumor and eye diseases, and as pro-VEGF therapy, to stimulate blood vessel growth for ischemia and wound healing in different pathological processes. These two opposite strategies of using VEGF therapy have many clinical applications in wet age-related macular degeneration, diabetic retinopathy and macular edema, retinal vein occlusion and in oncology (as monoclonal antibody or tyrosine kinase inhibitors), in ischemic heart, peripheral artery disease and treatment of chronic wound [21,22].

### 2.2. Placental Growth Factor (PlGF) in Placental Dysfunction

PIGF is a protein produced by the placenta that helps the growth of new blood vessels. It is primarily synthesized by syncytiotrophoblasts and villous trophoblasts within the placenta. PIGF is encoded by the PGF gene and its production is regulated by cellular differentiation, second messengers like cAMP and environmental stressors such as hypoxia [23,24]. It is synthesized in placental tissue and the most responsible gene, PGF gene, is transcribed into mRNA, translated into precursor proteins in the endoplasmic reticulum and assembled into glycosylated dimeric proteins before being secreted to stimulate angiogenesis. PGF gene is located on chromosome 14 and all the processes of transcriptions are controlled by promoter regions. Also, hypoxia-inducible factors, such as HIF-1alfa, can upregulate synthesis when oxygen levels drop. When the stimuli are present, fully formed packed PIGF dimers in form of vesicles could be secreted out of the cell, and it targets and bind to vascular endothelial growth factor receptor-1 (VEGFR-1) on endothelial cells [23,24].

PIGF is also a member of VEGF family and it is particularly relevant to placental vascular biology. PIGF contributes to angiogenesis by interacting primarily with VEGFR-1 and modifying VEGF-related signaling. In normal pregnancy, changes in PIGF concentrations are related to gestational age, reflecting the dynamic nature of placental vascular development. Placental dysfunction is characterized by reduction in circulating PIGF, and these reductions are usually related to the decreased production and/or increased sequestration by circulating sFlt-1. In clinical practice, isolated lower concentration of PIGF should not necessarily be interpreted as an isolated abnormality. Often, this situation is a part of a broader angiogenic imbalance with the impaired pro-angiogenic activity and higher anti-angiogenic signaling.

PIGF has two main clinical applications. It acts as critical biomarker for predicting and diagnosing pre-eclampsia in obstetrics and serves as a therapeutic target for managing tumor growth and wound healing in oncology and regenerative medicine [23,24,25].

### 2.3. Transforming Growth Factor-Beta (TGF-Beta) in Placental Dysfunction

TGF-beta is a broad acting protein signal that controls cell growth, tissue repair and immune response [26,27]. This protein is synthesized as an inactive precursor protein containing a signal peptide, a latency-associated peptide (LAP) and a mature TGF-beta domain. Initial synthesis is related to the cell translation of TGF-beta mRNA into a large precursor called pre-pro-TGF-beta. Then, the signal peptide is removed in the endoplasmic reticulum and starts to form a dimer linked by disulfide bonds. Interestingly, proprotein processing is in the Golgi apparatus and the next complex formation is translated into the small latent complex (SLC) to the final large latent complex (LLC). The final step is secretion, when the cell secretes the LLC into the extracellular matrix (ECM) in an inactive state. The latent transforming growth factor beta complex is activated in ECM through several distinct chemical, mechanical and enzymatic mechanisms. The most prominent physiological mechanism is related to the TGF-beta-1 and TGF-beta-3 isoforms. Besides that, chemical activation by changing the pH of the environment or high oxidative stress are important pathways which can denature the latent complex [26,27].

The clinical application of transforming growth factor beta (TGF-beta) is a major focus of modern drug development, primarily focused on inhibiting overactive TGF-beta signaling. TGF-beta therapeutics could be effective in early stages of tumor disease as a tumor suppressor by enforcing cell cycle arrest and maintaining tissue homeostasis. Paradoxically, these drugs could be harmful because of promoting tumor progression, driving the metastasis via epithelial-to-mesenchymal transition (EMT) and suppressing the immune system [26,27,28].

### 2.4. Soluble fms-like Tyrosine Kinase-1 (sFlt-1) in Placental Dysfunction

Soluble fms-like tyrosine kinase-1 is a protein capable of stopping new blood vessels from growing [29]. On a molecular level, sFlt-1 blocks the VEGF and PIGF and comes mostly from the placenta during pregnancy. This protein is synthesized primarily by the placenta by alternative mRNA splicing of the vascular endothelial growth factor receptor-1 gene (FLT1) and it acts as a decoy receptor that binds and neutralizes free VEGF and PIGF. The exact mechanism of synthesis is based on three facts: alternative splicing, protein structure and cellular origin. The FLT1 gene undergoes alternative pre-RNA processing to omit the transmembrane and intracellular signaling domains. The resulting sFlt-1 protein contains only the extracellular ligand-binding domain which lacks the capacity to induce the tyrosine kinase phosphorylation. Soluble fms-like tyrosine kinase-1 is mostly secreted by placental trophoblast cells into the maternal circulation, and with a small contribution from endothelial cells and monocytes during stress reaction [30,31].

In placental dysfunction, increased placental production of sFlt-1 may occur in response to hypoxia, oxidative stress and other forms of placental cellular stress. In a biochemical sense, sFlt-1 reduces the circulating levels of VEGF and PIGF which leads to endothelial dysfunction, increased vascular permeability, hypertension, renal endothelial injury, proteinuria and pulmonary or systemic manifestations of pre-eclampsia [29,30,31].

Clinical application of sFlt-1 is very significant, since the detection of sFlt-1 is followed by the angiogenic imbalance which develops before the clinical presentation of pre-eclampsia. So, the main application of sFlt-1 lies in the pre-eclampsia prediction (high sFlt-1 to PIGF ratio helps to predict and diagnose pre-eclampsia weeks before severe symptoms appear); in rule-out value (when the sFlt-1/PIGF ratio safely rules out the short-term development of pre-eclampsia) and in fetal monitoring (elevated levels of sFlt-1 correlate with placental dysfunction and fetal growth restriction) [29,30,31].

### 2.5. Soluble Endoglin (sEng) in Placental Dysfunction

Soluble endoglin is a circulating protein released by the placenta and damaged blood vessels [32]. The main role of its anti-angiogenic activity is to stop new blood vessel growth. Also, it has a significant role in causing high blood pressure and organ injury during severe pregnancy conditions such as pre-eclampsia and eclampsia. Soluble endoglin is synthetized when cell-surface endoglin is cut by specific enzymes like matrix metalloproteinase-14 (MMP-14). This proteolytic cleavage releases the large outer portion of the protein into the bloodstream as sEng, where it acts as an anti-angiogenic factor [33]. The exact mechanism of sEng synthesis is based on base protein expression and enzymatic shedding. The full-length endoglin gene is transcribed in vascular endothelial cells and syncytiotrophoblasts, regulated by factors like TGF-beta and HIF-1. In this process, enzyme MMP-14 targets the membrane-bound endoglin protein near the cell surface. After that, it is released into circulation as soluble endoglin (sEng) [32,33].

Many states could induce secretion of this protein and regulate further production. Hypoxia and elevated oxidative stress in tissues stimulate production of reactive oxygen species and cellular stress. These stress signals increase the activity of MMO-14 while reducing tissue inhibitors of metalloproteinases such as TIMP-3, accelerating the cutting process. In an abnormal placenta, present high local or systemic stress is present, and that is the reason for higher sEng release, contributing to widespread blood vessel damage and clinical symptoms like elevated arterial blood pressure in pregnant women [32,33].

In clinical practice, sEng is mechanistically complementary to sFlt-1. Whereas sFlt-1 predominantly interferes with VEGF/PIGF signaling, sEng affects pathways involved in endothelial homeostasis and vascular tone. Based on that, clinical evidence suggests that increased sEng may act synergistically with sFlt-1, particularly in severe or early-onset disease. Specifically, elevated sEng levels in maternal serum often combined with the sFlt-1/PIGF ratio serve as a powerful biomarker in predicting and diagnosing pre-eclampsia and HELLP syndrome, as a tool for cardiovascular risk assessment and for monitoring vascular pathologies such as cerebral arteriovenous malformations and tumor angiogenesis [32,33,34]. Table 1 presents a comparison of sFlt-1 and sEng in structural, biochemical and clinical diagnostic profile.

## 3. Divergent Pathophysiological Pathways: Shallow vs. Hyper-Invasive Placentation

To clarify the overarching concept of “abnormal placentation,” a fundamental distinction must be made between disorders of ischemic placental insufficiency and disorders of hyper-invasive placentation. Although preeclampsia, fetal growth restriction (FGR), and Placenta Accreta Spectrum (PAS) all fall under the broad clinical umbrella of abnormal placentation, they represent opposite ends of the pathophysiological spectrum and are not interchangeable manifestations of the same trophoblastic abnormality [35,36,37].

Shallow Placentation: Preeclampsia and FGR: The pathogenesis of preeclampsia and early-onset FGR is defined by under-invasion or shallow invasion of the maternal decidua and myometrium by extravillous trophoblasts (EVTs). Under physiological conditions, EVTs successfully remodel the maternal spiral arteries from high-resistance, low-capacity vessels into dilated, low-resistance conduits. In preeclampsia and FGR, this process fails. The maternal vessels remain highly restrictive, culminating in chronic placental hypoxia, fluctuating perfusion, and ischemia–reperfusion injury. At the molecular level, this ischemic stress triggers an “angiogenic switch,” forcing the syncytiotrophoblast to overproduce anti-angiogenic factors (sFlt-1 and sEng) while suppressing pro-angiogenic molecules (PlGF) [35,36,37].

Hyper-Invasive Placentation: Placenta Accreta Spectrum (PAS): In stark contrast, PAS is fundamentally characterized by an abnormal, hyper-invasive adherence of the placenta to or through the uterine wall. The primary defect in PAS is not intrinsic to the trophoblast itself, but rather stems from a deficient or completely absent decidua basalis, typically caused by prior iatrogenic uterine trauma (such as previous Cesarean deliveries, curettage, or myomectomy) [35,36].

Lacking the protective biological barrier normally provided by the Nitabuch’s layer and the decidua, the EVTs encounter no physiological resistance. As a result, they invade deeply into the myometrium (placenta increta) or breach the uterine serosa into adjacent pelvic organs (placenta percreta).

Treating these distinct disorders as part of the same mechanistic continuum obscures their unique etiologies. In summary, preeclampsia and FGR represent a failure to establish adequate maternal blood supply due to insufficient invasion, whereas PAS represents a catastrophic structural breach of the uterine compartments due to an absent decidual boundary.

## 4. Angiogenic Imbalance as a Consequence of Abnormal Placentation

**Figure 1:**
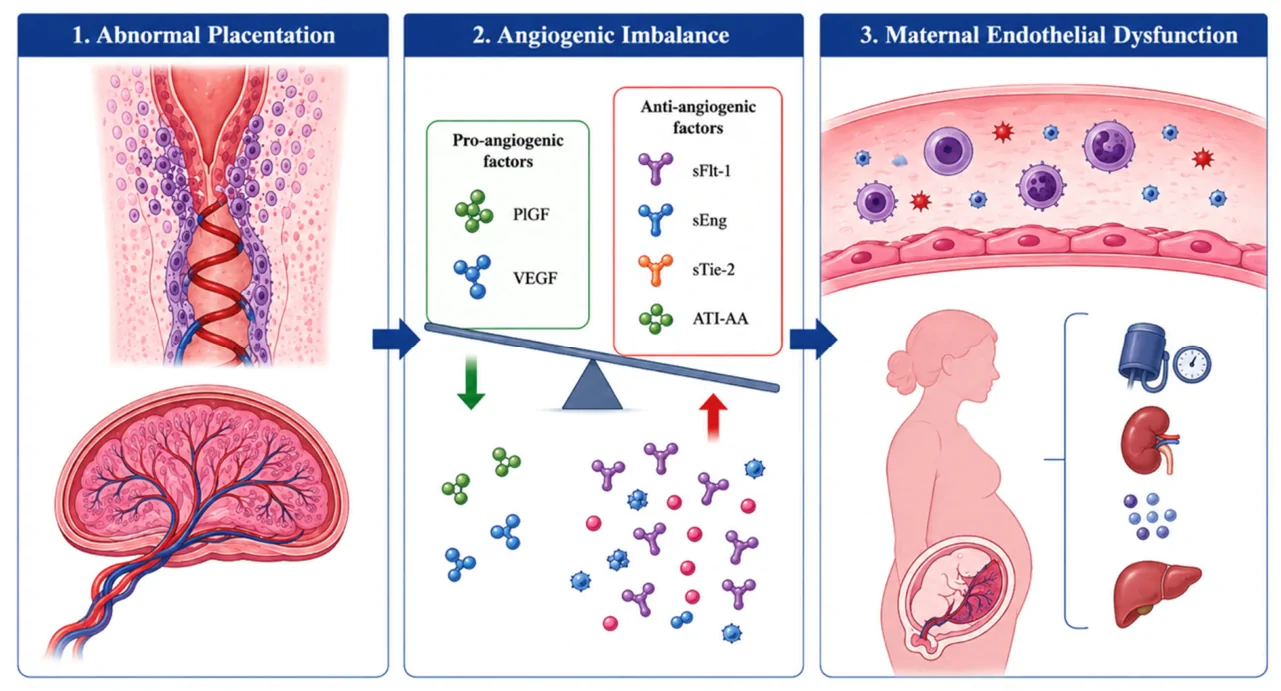
Mechanism of placental dysfunction: molecular overview [35,36,37]. (1) Abnormal placentation: inadequate extravillous trophoblast invasion and impaired spiral-artery remodeling result in a high-resistance uteroplacental circulation and reduced placental perfusion. (2) Angiogenic imbalance: placental hypoxia and cellular stress alter the balance between pro-angiogenic factors, including placental growth factor (PlGF) and vascular endothelial growth factor (VEGF), and anti-angiogenic factors, including soluble fms-like tyrosine kinase-1 (sFlt-1), soluble endoglin (sEng), soluble Tie-2 (sTie-2), and angiotensin II type-1 receptor agonistic autoantibodies (AT1-AA). Increased sFlt-1 sequesters PlGF and VEGF, reducing their bioavailability and impairing endothelial pro-angiogenic signaling. (3) Maternal endothelial dysfunction: the resulting angiogenic imbalance promotes endothelial activation, inflammation, oxidative stress, vasoconstriction, and increased vascular permeability, contributing to hypertension, proteinuria, renal and hepatic dysfunction, and adverse fetal outcomes such as fetal growth restriction and preterm birth. Abbreviations: AT1-AA, angiotensin II type-1 receptor agonistic autoantibodies; PlGF, placental growth factor; sEng, soluble endoglin; sFlt-1, soluble fms-like tyrosine kinase-1; sTie-2, soluble Tie-2; VEGF, vascular endothelial growth factor.

The development of placental dysfunction is generally understood as a multifactorial and multistep process [35]. The literature evidence most often supports the three-step process of placenta dysfunction [35,36]. The exact order and mechanism of placental dysfunction is graphically presented in Figure 1.

The second classification is based on the four phases of placental dysfunction [36]. The first phase involves abnormal placentation or placenta accrete sprectrum (PAS). Abnormal placentation occurs when fetal trophoblast cells invade the maternal uterine wall improperly, resulting in eihter shallow invasion or excessive invasion via disrupted molecular signaling. Extravillous trophoblast (EVTs) fail to switch from an epithelial to an endothelial phenotype, lacking the necessary adhesion molecules like integrins (alfa1betaa and VE-cadherin). The next process is related to the inadequate spiral artery remodeling, when trophoblasts fail to replace the muscular and elastic layer of maternal spiral arteries, keeping them high-resistance and low flow. Angiogenic imbalance is a very important factor which results in overexpression of anti-angiogenic factors like sFlt-1, which binds and neutralizes pro-angiogenic proteins VEGF and PIGF. In addition, immune maladaptation could be developed, such as mismatch between maternal uterine natural killer (uNK) and fetal human leukocyte antigens (HLA-C) resulting in reduced normal trophoblast migration. In abnormal placentation, the evidence supports the significance of type-1 epithelial-to-mesenchymal transition (EMT) as the most important signaling pathway. Dysregulation of key upstream signaling pathways disrupt EMT, leading to insufficient invasion seen in pre-eclampsia or the uncontrolled over-invasion seen in placenta accrete spectrum (PAS). Table 2 presents the key signaling pathways involved in abnormal placentation. As a result, the uteroplacental circulation remains relatively high resistance [36,37].

The second stage in abnormal placentation is oxidative stress [35]. Reduced or fluctuating placental perfusion may promote hypoxia, ischemia–reperfusion injury, oxidative stress, mitochondrial dysfunction and inflammatory signaling. Oxidative stress is the fundamental bridge linking early inadequate placental development to systemic maternal disease. When the production of ROS overloads the local antioxidant defense system, oxidative stress starts. It is known that the trigger event is ischemic –reperfusion injury, probably because of the mechanical cause (incomplete remodeling of maternal spiral arteries), intermittent blood flow (intermittent turbulent flow) or mitochondrial damage (disrupted electron transport chain causing electrons to leak out and react with molecular oxygen). In this process of oxidative stress mediated injury, specific enzymes are included, such as NADPH oxidase, xanthine oxidase and uncoupled nitric oxide synthase (eNOS). When the oxidative stress and endoplasmic reticulum reach a critical threshold, the outer layer of placenta-the syncytiotrophoblast, begins to break apart.

In a biochemical sense, the structural integrity and function, the placenta depends on a balance between pro-angiogenic factors, which promote blood vessels growth and remodeling, and anti-angiogenic factors, which inhibit vessel growth and cause endothelial dysfunction. During a normal pregnancy, pro-angiogenic factors dominate early on to facilitate low-resistance, high-flow blood delivery to the fetus. In placental dysfunction, such as in pre-eclampsia and fetal growth restriction, this balance is impaired and reversed. Anti-angiogenic factors shift into overdrive, neutralizing pro-angiogenic molecules and causing widespread vascular damage. Table 3 presents biological roles, molecular pathways and status of all angiogenic factors in placental abnormality (Table 3). This imbalance of angiogenesis is a third stage of this process of abnormal placentation. The final stage is maternal systemic endothelial dysfunction, which produces the clinical manifestation of pre-eclampsia [41,42].

## 5. Variations in Features and Angiogenic Markers in Placental Insufficiency

Although preeclampsia and fetal growth restriction share a common pathophysiological substrate rooted in angiogenic imbalance, they represent highly heterogenous clinical entities. Understanding the distinct molecular and clinical phenotypes is essential for accurate risk stratification and targeted management [43,44]. Table 4 presents differences between the clinical phenotypes of placental dysfunction (Table 4).

## 6. Molecular Mechanisms and Angiogenic Factors in Early Pregnancy

Early pregnancy and placentation (placental formation) involve complex embryo-maternal molecular crosstalk [43]. The unique process is trophoblast differentiation under the hormone production and invasive anchoring. Then starts vascular remodeling of maternal spiral arteries, when the endothelial cells start to be replaced with extravillous trophoblasts. Also, immunological changes and oxidative regulation take a significant role in early placentation. The key-forming placenta biomarkers are pregnancy-associated plasma protein-A (PAPP-A), placental growth factor (PIGF), soluble fms-like tyrosine kinase-1 (sFlt-1), placental protein 13 (PP13) and microRNAs (miRNAs), which circulate in maternal blood and reflect early trophoblast stress or proliferation [44,45].

Successful placentation requires a precise balance between embryonic invasion and maternal tissue remodeling. It transforms high-resistance maternal blood vessels into low-resistance, high flow channels to ensure adequate nutrient and oxygen exchange for the fetus. Controlled trophoblast invasion is very important, and it is presented in invasion of EVTs through the uterine lining (decidua) and into the inner third of the muscular uterine wall (myometrium). “The vascular switch” is a name for complex process of spiral artery remodeling [46]. For the first, EVTs replace the maternal endothelial and smooth muscle cells of the uterine arteries, then EVTs switch their surface receptors to vascular type and finally hemodynamic changes start (low resistance and velocity). Before 10 gestational weeks, placentation works in a low oxygen environment (hypoxic, ~2–3% O2), which protects the embryo from oxidative damage and keeps trophoblasts in a highly proliferative state. In the period between 10 and 12 weeks, oxygen rises and trophoblasts stop dividing and become deeper. In this period, uterine natural killer (uNK) cells recognize HLA markers (A or B) and secrete growth factors such as VEGF and IL-8 that help remodel the maternal blood vessels. In detail, the process of early placentation through the role of molecular factors is presented in Table 5 [45,46,47].

## 7. Clinical Application of Angiogenic Factors in Predicting Placental Dysfunction

The clinical application of angiogenic factors represents a monumental shift in modern perinatology [48]. It transitions management from reactive symptom control to proactive objective prediction and risk stratification of placental dysfunction, primarily pre-eclampsia and fetal growth restriction.

### 7.1. The Angiogenic Index: sFlt-1/PIGF Ratio

This ratio represents a clinical tool for assessing placental health as a ratio between anti-angiogenic sFlt-1 and pro-angiogenic PIGF [49]. In normal pregnancy, PIGF levels rise during the first two trimesters while sFlt-1 remains low. When placental dysfunction occurs, this balance shifts extremely before physical symptoms emerge (hypertension and proteinuria). In clinical practice, an sFlt-1/PIGF ratio less than 38 has negative predictive values, exceeding 99%, which means that the patient will not develop pre-eclampsia within the next 1 to 4 weeks. The value higher than 85 indicates an extremely high risk of developing pre-eclampsia and placental dysfunction outcomes within 4 weeks [49,50].

### 7.2. The Combined Test Screening

In early pregnancy before 13 weeks of gestation, PIGF testing is integrated into predictive risk assessment (the Fetal Medicine Foundation or FMF algorithm). This screening tool means that the levels of PIGF are combined with the maternal history, mean arterial pressure (MAP), and the uterine artery pulsatility index (UtA-PI) measured via Doppler ultrasound. Sometimes, in clinical settings, the measurement of blood pressure and proteinuria can be confounding, but the measuring with the sFlt-1/PIGF ratio allows to distinguish between chronic hypertension and gestational hypertension [51,52]. In clinical practice, different variations are observed. Inter-assay variations and lack of interchangeability are important limitations, where the values of sFlt-1/PIGF cut-offs are platform specific and strongly depend on technologies and manufacturers. Also, gestational-age dependence is very often present, and the levels of sFlt-1 rise gradually. The baseline distribution of angiogenic factors is influenced by genetic background, maternal comorbidities, and socioeconomic factors, such as Black or African American women exhibit slightly lower median of this ratio compared with White women, than comorbidities like gestational diabetes mellitus (GDM) or chronic kidney disease (CKD) can independently modulate circulating angiogenic components. Definitely, the clinical implementation of the soluble fms-like tyrosine kinase-1 to placental growth factor (sFlt-1/PlGF) ratio is highly dynamic and must be observed within a specific clinical context.

### 7.3. Prediction of Fetal Growth Restriction

Placental insufficiency directly impairs the delivery of vital nutrients and oxygen to the fetus [53]. Molecular cascade of angiogenic control in placental homeostasis and fetal growth restriction is very complex. In normal pregnancy, proper extravillous trophoblast invasion completely transforms maternal spiral arteries into wide, low-resistance, high-capacitance conduits. This process maintains normal placental oxygenation (normoxia). Under these physiologic conditions, the placenta synthesizes and secretes balanced amounts of pro-angiogenic factors: vascular endothelial growth factor (VEGF) and placental growth factor (PLGF). These free-floating signaling ligands dock directly into their respective transmembrane receptors (VEGFR-1/Flt-1 and KDR/Flk-1) situated on the surface of endothelial cells. Receptor dimerization triggers standard intracellular signal transduction pathways that upregulate endothelial nitric oxide synthase (eNOS). The resulting release of Nitric Oxide (NO) promotes maternal-fetal vasodilation, vascular stability, and optimal villous capillary branching, establishing a healthy physiological baseline for normal fetal development [53,54,55].

In abnormal pregnancy and during fetal growth restrictions, defective trophoblasts invade and leave maternal spiral arteries un-remodeled, keeping it very narrow, rigid and highly resistant [56]. This is the reason for placental ischemia and hypoxia. Low oxygen tension stabilizes the alpha subunit of hypoxia-inducible factor 1 (HIF-1α), preventing its degradation and driving its translocation into the nucleus. This transcriptionally upregulates the synthesis and systemic release of anti-angiogenic decoy proteins: soluble fms-like tyrosine kinase-1 (sFlt-1) and soluble endoglin (sEng). Once in circulation, sFlt-1 functions as a molecular “decoy sponge,” physically binding and trapping free VEGF and PLGF, which prevents them from reaching the cell membrane. Concurrently, sEng impairs local transforming growth factor beta (TGF-β) pathways. This molecular trapping leaves native endothelial membrane receptors vacant, completely suppressing the downstream eNOS activation cascade. The loss of nitric oxide homeostasis causes localized endothelial starvation, severe vasoconstriction, and restricted vascular branching, directly limiting nutrient transport and causing fetal growth restriction [55,56].

Based on previous explanations, measuring the PIGF and sFlt-1/PIGF ratio could be strongly helpful in predicting fetal growth restriction. When FGR persists, PIGF levels will be low and the sFlt-1/PIGF ratio will be elevated. International guidance has recognized the potential value of angiogenic biomarkers in women with suspected hypertensive disorders. The 2021 ISSHP recommendations describe reduced PlGF or an elevated sFlt-1/PlGF ratio as evidence of angiogenic imbalance and uteroplacental dysfunction, while emphasizing variability between studies [57,58]. Nevertheless, thresholds should not be interpreted universally because assays, gestational ages, populations, and clinical indications differ [57,58].

## 8. New Angiogenic Factors and Epigenetic Implications

Placentation relies on a precise balance of pro- and anti-angiogenic factors to build the placental vascular network. Beyond traditional pathways such as VEGF, new potential factors include prokineticins, endocrine gland-derived endothelial growth factor (EG-VEGF), netrins and Hedgehog signaling proteins [59,60].

Prokineticins such as PROK1 and PROK2 are newly characterized pro-angiogenic factors which could regulate tight trophoblast proliferation, differentiation and vascular permeability in the decidua. These factors have a significant role in early pregnancy that regulate primarily uterine receptivity, implantation and placental vascular development. PROK1 is expressed heavily in the placenta, decidua and steroidogenic tissues and circulating and localized levels rise dramatically during the first trimester with the peak between 8 and 10 weeks of gestation. PROK2 is predominantly expressed within the central nervous system, driving the development of gonadotropin-releasing hormone (GnRH) neurons. Interestingly, its peripheral expression in the placenta is minimal, so its direct role in pregnancy remains questionable [59,60].

EG-VEGF selectively targets endocrine gland endothelium to promote vessel growth and support early placental villous morphogenesis. In the literature, this marker is scientifically cataloged as PROK1, but EG-VEGF acts selectively at the maternal-fetal interface to drive early anatomical foundation of human placentation. In early gestation, this marker arises with the peak between 8 and 11 of pregnancy (~250 pg/mL), before declining back to baseline.

Netrins are characterized as pro-angiogenic also, and known as neuronal guidance cues, which have distinct role in promoting endothelial cell recruitment and vascular branching. They represent a family of secreted proteins originally identified as axon guidance cues in the central nervous system, but they are now recognized as essential vascular guides in the cardiovascular system and placental development. In some articles, netrins are characterized as a factor with dual angiogenic activity. The previous evidence suggested two types of netrins, 1 to 4, which exert biological effects by binding to specific cell-surface receptors. Preclinical studies confirmed, but clinical relevance is still not fully recognized, and the netrins are imbalanced in preeclamptic pregnancies and especially in fetal growth restrictions, where the disrupted netrin signaling directly impairs the branching of villous capillaries.

Hedgehog proteins [61], also proposed as pro-angiogenic, modulate extracellular matrix breakdown and pericyte recruitment for vessel stability. Their main differences are based on role and activity, netrin-1 is primarily pro-angiogenic in early pregnancy, while the netrin-4 has an inhibitory role often. Unlike VEGF, which drives the initial sprouting of fragile capillaries, Hedgehog signaling is responsible for structural maturation. The loss of Hedgehog signaling leads to a lack of pericyte coverage, causing the placental villous capillaries to become structurally unstable, hyper-permeable and prone to premature degeneration, which strongly contributes to high clinical application. In animal models, Hedgehog agonists are tested to rescue placental function, and have suggested a new specific epigenetic mechanisms like DNA methylation which are able to reduce Hedgehog proteins in pre-eclampsia [61,62].

As anti-angiogenic intermediates, in the preclinical research literature are suggested microRNAs [63], as post-transcriptional regulators that fine-tune expression profiles of local pro- and anti-angiogenic signals and SGK1 pathways, which intermediates modulating PIGF-mediated cross-talk between endometrial stromal cells and endothelial cells.

The clinical application of newly characterized angiogenic markers represents a major paradigm shift in modern obstetrics. All the factors are categorized in two groups: established gold-standards (sFlt-1, PIGF, sFlt-1/PIGF ratio), which are widely approved and actively integrated in clinical guidelines, and emerging novel markers (sEng, microRNA, VEGFR-2, adrenomedullin), which are needed for prognostic research and diagnostic trials to confirm their clinical significance.

## 9. Limitation of Current Evidence

While angiogenic markers such as the sFlt-1/PIGF ratio have revolutionized risk stratification for placental dysfunction, significant limitations persist in current clinical and molecular evidence [11,64]. The main gaps lie in the scientific evidence and therapeutic implementation regarding the angiogenic factors in placental dysfunction. In detail, poor acute prognosis is something that limits the prediction of maternal complications. Clinical evidence demonstrates that the sFlt-1/PIGF ratio is a poor predictor for specific maternal adverse events once pre-eclampsia is diagnosed. Definitely, Elevated sFlt-1 or reduced PlGF may occur in different forms of placental dysfunction and should therefore be interpreted in a clinical context, and very important, concentrations are strongly influenced by gestational age and maternal characteristics.

Large cohort trials show [64] that angiogenic profiles fail to provide predictive or diagnostic value for fatal complications such as postpartum hemorrhage, placental abruption or maternal stroke. Furthermore, the biological processes underlying early placental dysfunction are heterogeneous. Not all cases of preeclampsia or FGR are driven by identical mechanisms. For example, current evidence and guidelines do not perfectly adjust for physiological variations, such as body mass index, chronic kidney disease and other maternal characteristics, which can lead to false-positive or false-negative interpretations [64,65].

For potential new anti-angiogenic factors, the literature data are primarily associative or observational. Large-scale longitudinal clinical data are needed to establish a causative link between angiogenic factors and early-pregnancy failure. It is clear that an abnormal angiogenic profile may represent an early pathogenic signal, but this claim requires further investigations.

## 10. Future Directions

The future of angiogenic factors in pregnancy lies in transitioning from purely prognostic markers to targeted therapeutics and personalized precision medicine. Currently, studies are focused on overcoming the limitations of late-stage diagnosis by correcting angiogenic imbalance in real time to safely prolong gestation and improve neonatal outcomes. Future directions must include selective siRNA silencing, nanoparticle targeting, additional biomarkers and machine learning algorithms. In addition, future guidelines for predicting placental dysfunction should incorporate the potential of drugs already in clinical practice, such as statins or metformin, which are effective in reducing antiangiogenic toxins from the hypoxic placenta, offering a low-cost preventive option for high-risk populations. Furthermore, current guidelines should focus on standardization and global reference ranges, in order to establish an international standardized database of angiogenic profiles across ethnic groups and multifetal gestations. In addition, experimental and early translational approaches should include and identify the best strategies aimed at reducing circulating sFlt-1 or restoring proangiogenic signaling, although these approaches remain in the exploratory phase. When evaluating the clinical translation of newly characterized angiogenic and placental molecules, it is vital to apply a strict hierarchy of clinical evidence. A common pitfall in biomarker research is the rapid extrapolation of statistical correlation into clinical utility or therapeutic action. To ensure translation fidelity, four distinct developmental tiers must be systematically decoupled: pathophysiological association, independent predictive performance, clinical utility and therapeutic evidence (rather than identifying a molecule only as a central actor in pathogenesis and taking all the characteristics into the account).

## 11. Conclusions

This narrative review comprehensively presented information regarding all characteristics of angiogenic factors in early pregnancy and placentation, which is a prerequisite for a healthy pregnancy and a healthy fetus. Angiogenesis is fundamental to normal placental development, and disruption of the physiological balance between pro-angiogenic and anti-angiogenic mediators represents an important mechanism in early placental dysfunction.

Angiogenic factors play a central role in the pathophysiology of placental dysfunction and provide an important molecular link between abnormal placentation and maternal endothelial disease. VEGF and PlGF promote vascular development and endothelial homeostasis, whereas sFlt-1 and sEng act as important anti-angiogenic mediators. Excessive sFlt-1 reduces the bioavailability of VEGF and PlGF, while sEng interferes with endothelial signaling pathways. Together, these abnormalities contribute to maternal endothelial dysfunction and the clinical manifestations of placenta-mediated disease.

The angiogenic imbalance may precede the clinical presentation of preeclampsia and may also be associated with fetal growth restriction. Among currently available biomarkers, the sFlt-1/PlGF ratio has the strongest clinical evidence for risk stratification in women with suspected preeclampsia.

Nevertheless, the role of angiogenic factors in the earliest stages of placental dysfunction remains incompletely defined. The major challenge is to determine whether angiogenic abnormalities can identify clinically meaningful placental dysfunction early enough to permit effective intervention. This suggests that pro-angiogenic therapeutics could be a possible option for the treatment of pre-eclampsia, but also a more specific and sensitive predictive and diagnostic tool in modern obstetrics.

## Figures and Tables

**Table 1:** Comparison of sFlt-1 and sEng in structural, biochemical and clinical diagnostic profile.

Feature	sFlt-1 (Soluble fms-like Tyrosine Kinase-1)	sEng (Soluble Endoglin)
Chemical Structure	Soluble homodimeric glycoprotein fragment Lacks transmembrane and cytoplasmic domains Composed of 6 extracellular Ig-like domains (binds VEGF and PlGF)	Soluble truncated homodimeric glycoprotein fragment Cleaved extracellular domain of membrane endoglin Composed of orphan and zona pellucida (ZP) domains (binds TGF-β1/3)
Synthesis	Produced via alternative splicing of the FLT1 gene mRNA Splicing drops exon 13-18 transcripts, omitting the anchor sequence	Produced via proteolytic cleavage of membrane-bound endoglin (ENG) Cleavage mediated heavily by matrix metalloproteinase-14 (MMP-14)
Place of Release	Primary: Hypoxic placental syncytiotrophoblasts Secondary: Endothelial cells and monocytes, entering systemic circulation	Primary: Ischemic placental syncytiotrophoblasts Secondary: Endothelial cell surfaces during vascular stress
Laboratory Range & Units	14–20 + 6 g.w. Normal pregnancy: 1000–1800 pg/mL; Preeclampsia: >2500 pg/mL 21–28 + 6 g.w. Normal pregnancy 1200–2800 pg/mL Preeclampsia: >4500–6500 pg/mL 29–33 + 6 g.w. Normal pregnancy 1500–3500 pg/mL Preeclampsia: >6000–12,000 pg/mL 34–40 + 6 g.w. Normal pregnancy 2500–5500 pg/mL Preeclampsia: >10,000–25,000 pg/mL Twin pregnancies: 1.5- to 2-fold times higher BMI > 30 kg/m^2^: 15–20% reduction of sFlt-1 Attention: lower baseline can mask early-stage preeclampsia	14–20 + 6 g.w. Normal pregnancy: 3000–3500 pg/mL; Preeclampsia: >7000 pg/mL 21–28 + 6 g.w. Normal pregnancy 4000–6000 pg/mL Preeclampsia: >10,000 pg/mL 29–33 + 6 g.w. Normal pregnancy 4500–7500 pg/mL Preeclampsia: >15,000 pg/mL 34–40 + 6 g.w. Normal pregnancy 6000–9500 pg/mL Preeclampsia: >25,000–40,000+ pg/mL
Sensitivity	80% to 85% individually for predicting preeclampsia development within 4 weeks Rises to ~95% sensitivity when evaluated as the sFlt-1/PlGF ratio	75% to 82% individually for standard onset Higher sensitivity (~90%) for identifying early-onset preeclampsia and severe HELLP syndrome variants
Specificity	88% to 92% individually for rule-out within 1 week Rises to 94% to 96% when integrated into the sFlt-1/PlGF clinical ratio calculation	85% to 90% individually Provides highly specific secondary verification when paired with sFlt-1 to reduce false-positive rates

**Table 2:** Molecular signaling pathways in abnormal placentation [35,36,37,38,39,40]. Abbreviations: SNAIL: Snail Family Transcriptional Repressor 1, SLUG: Snail Family Transcriptional Repressor 2, TWIST: Twist Family BHLH Transcription Factor 1, ZEB1/ZEB2: Zinc Finger E-Box Binding Homeobox 1 and 2, CDH1: Cadherin 1/E-cadherin, CDH2: Cadherin 2; N-cadherin; TGF-β1, 2, 3: transforming growth factor beta 1, 2, and 3; Wnt3a/Wnt5a: Wingless-Type MMTV Integration Site Family Members 3A and 5A; LRP5/LRP6: LDL Receptor-Related Protein 5 and 6; EGF: Epidermal Growth Factor, IGF-1/IGF-2: Insulin-like Growth Factors 1 and 2; HGF: Hepatocyte Growth Factor; VEGF: vascular endothelial growth factor; DLL1/DLL4: Delta-Like Canonical Notch Ligand 1 and 4; Smad2, 3, 4: Mothers Against Decapentaplegic Homologs 2, 3, and 4; HES1: Hes family bHLH transcription factor 1; HEY1: Hes related family bHLH transcription factor with YRPW motif 1; SNAIL1, SLUG2: mTORC1/mTORC2: Snail family transcriptional repressor 1 and 2; Mechanistic Target of Rapamycin Complex 1 and 2; GSK-3β: Glycogen Synthase Kinase-3 Beta, TCF/LEF: T-Cell Factor/Lymphoid Enhancer Factor; PI3K: Phosphoinositide 3-Kinase; MMP-2, 7, 9: matrix metalloproteinases 2, 7, and 9; sFlt-1: soluble fms-like tyrosine kinase-1; sEng: soluble endoglin; PE is preeclampsia; PAS: Placenta Accreta Spectrum; FGR: fetal growth restriction.

Signaling Pathway	Key Upstream Signals & Ligands	Downstream Markers & Target Genes	Primary Place of Action/Subcellular Site	Associated Pathology
TGF-b/Smad Pathway	TGF-β1, TGF-β2, TGF-β3, Activins, Nodal	Smad2/3/4, SNAIL, SLUG, TWIST, ZEB1/2, downregulates E-cadherin (CDH1)	Cytotrophoblasts & Extravillous Trophoblasts (EVTs); Nucleus & Cytoplasm	PAS (Hyper-activated)/PE (Impaired signaling)
Canonical Wnt/b-Catenin	Wnt3a, Wnt5a, Frizzled receptors, LRP5/6, GSK-3β inhibitors	Stabilized b-catenin, TCF/LEF transcription factors, N-cadherin (CDH2), MMP-7	Anchoring villi, invasive front of EVTs; Cell–cell junctions to Nucleus	PE & FGR (Downregulated)/PAS (Upregulated)
PI3K/Akt/mTOR	EGF, IGF-1, IGF-2, HGF, VEGF, Angiopoietins	Phospho-Akt, mTORC1/mTORC2, MMP-2, MMP-9, Vimentin, Cyclin D1	EVTs invading interstitial uterine matrix; Cytoplasm & Plasma membrane	PAS (Over-activated)/Severe early-onset PE (Suppressed via hypoxia)
Notch Signaling	Jagged1/2, Delta-like ligands (DLL1, DLL4), Notch1–4 receptors	NICD (Notch Intracellular Domain), HES1, HEY1, SNAIL1, SLUG	Maternal-fetal contact zone, endothelial-EVT interaction site; Membrane to Nucleus	PE & Shallow Invasion (Deficient signaling/locked phenotypes)
HIF-1a/Hypoxia Axis	Low oxygen tension (<2% O2), Cobalt chloride mimicking factors	HIF-1a, sFlt-1 (soluble fms-like tyrosine kinase-1), sEng (soluble endoglin), VEGF	Early first-trimester anchoring villi, syncytiotrophoblast layer; Nucleus	Preeclampsia (Pathologically sustained high expression via chronic hypoxia)

**Table 3:** Comprehensive overview of pro-angiogenic and anti-angiogenic factors in placental dys (function) [41,42].

Factor & Type	Biological Significance	Molecular Pathway & Mechanism	Status in Normal Placenta	Status & Clinical Application in Dysfunction
Pro-angiogenic factors				
PLGF (Placental Growth Factor)	Regulates healthy placental angiogenesis, trophoblast proliferation, and maternal spiral artery remodeling.	Binds selectively to VEGFR-1 (Flt-1). Directly drives downstream endothelial nitric oxide synthase (eNOS) activation and nitric oxide (NO) secretion.	Highly expressed during early and mid-pregnancy; peaks between weeks 28–32 to ensure low-resistance vascular channels.	Significantly Decreased. Low maternal serum PLGF levels serve as a primary early screening biomarker for FGR and preeclampsia before symptom onset.
VEGF (Vascular Endothelial Growth Factor)	Core driver of embryonic vasculogenesis, capillary vessel branching, and endothelial cell survival pathways.	Binds to both VEGFR-1 (Flt-1) and VEGFR-2 (Flk-1/KDR). Triggers intracellular MAPK/ERK and PI3K/Akt protective survival cascades.	Expressed at consistent baseline levels locally within tissue layers to preserve microvascular integrity.	Decreased Free Bioavailability. Active molecules are heavily scavenged and locked down by pathologically high levels of circulating decoy receptors.
TGF-β1 (Transforming Growth Factor-Beta 1)	Coordinates local cell differentiation, extracellular matrix development, and structural vascular tissue remodeling.	Signals through a heteromeric receptor complex containing ALK-1 or ALK-5, initiating internal Smad2/3 phosphorylation events.	Maintains a precise physiological balance during interstitial and endovascular trophoblast invasion mechanisms.	Dysregulated Signaling. Intracellular downstream pathways are interrupted by circulating soluble endoglin co-receptors, contributing to vasoconstriction.
Anti-angiogenic factors				
sFlt-1 (Soluble fms-Like Tyrosine Kinase-1)	Acts as a natural antagonist to pro-angiogenic activity; helps regulate over-exuberant vascular growth near full term.	Lacks a transmembrane structural domain. Circulates freely in blood to bind and sequester free VEGF and PLGF, acting as an ‘angiogenic sponge’.	Maintained at stable, low levels throughout early and mid-gestation; experiences a mild, physiologic rise immediately prior to labor.	Pathologically Upregulated. Extreme surges precede FGR clinical onset. Utilized worldwide via the maternal serum sFlt-1/PLGF ratio as a diagnostic and predictive metric.
sEng (Soluble Endoglin)	Inhibits capillary tube formation, induces endothelial structural breakdown, and drives systemic vascular narrowing.	Cleaved form of membrane-bound endoglin. Binds circulating TGF-β1 and TGF-β3, preventing them from docking to functional cell-surface receptors.	Expressed minimally during healthy placental development, supporting stable maternal blood pressure indices.	Significantly Elevated. Synergizes directly with elevated sFlt-1 to cause widespread maternal endothelial damage, placental insufficiency, and asymmetric FGR.

**Table 4:** Phenotypic heterogenity and angiogenic profiles in placental dysfunction.

Phenotype	Placental Pathology	Primary Doppler Signs	Angiogenic Profile (sFlt-1/PlGF)	Main Clinical Outcome
Early-Onset Preeclampsia (EO-PE)	Severe failure of extravillous trophoblast invasion and incomplete spiral artery remodeling; profound intrinsic placental disease.	Uterine artery bilateral notches, high resistance index (RI).	Extremely elevated (marked sFlt-1 upregulation and drastic PlGF suppression weeks before clinical onset).	Maternal hypertension and proteinuria presenting before 34 weeks of gestation; strong association with severe FGR.
Late-Onset Preeclampsia (LO-PE)	Mild placental involvement; primarily a maternal metabolic or cardiovascular mismatch where the placenta outgrows its capacity.	Often normal uterine and umbilical artery Doppler indices.	Normal to mildly/transiently elevated ratio; profiles remain closer to physiological values.	Maternal hypertension and proteinuria presenting at or after 34 weeks of gestation; usually normal fetal birth weight.
Early Fetal Growth Restriction (Early FGR)	High-grade villous hypoplasia, diffuse placental ischemia, and severe restriction of the functional maternal-fetal exchange surface.	Absent or reversed end-diastolic velocity (AEDV/REDV) in the umbilical artery; abnormal ductus venosus flow.	Significantly elevated ratio; severe local syncytiotrophoblast stress.	Fetal growth restriction presenting before 34 weeks of gestation; high risk of chronic fetal hypoxia and neonatal morbidity.
Late Fetal Growth Restriction (Late FGR)	Mild, focal, or localized placental lesions; placental structural capacity is only moderately compromised.	Often normal umbilical artery Doppler; characterized by brain-sparing redistribution (reduced cerebroplacental ratio—CPR).	Normal to only mildly elevated ratio; lower diagnostic sensitivity via maternal serum biomarkers.	Fetal growth restriction presenting at or after 34 weeks of gestation; high risk of acute intrapartum fetal distress.
Isolated Angiogenic Abnormality	Variable placental stress where angiogenic imbalance remains localized, or maternal endothelium possesses resilience against injury.	Variable; may demonstrate isolated uterine or umbilical artery resistance.	Significantly elevated sFlt-1/PlGF ratio or isolated profound suppression of PlGF without clinical signs of preeclampsia.	Elevated risk for normotensive FGR, abruptio placentae, idiopathic preterm birth, or adverse perinatal outcomes without maternal hypertension.

**Table 5:** Molecular biomarkers and angiogenic factors in early pregnancy. Abbreviations: VEGF and VEGF-A- vascular endothelial growth factor and vascular endothelial growth factor; AVEGFR-1 and Flt-1-vascular endothelial growth factor Receptor 1; PlGF: placental growth factor; LIF: Leukemia Inhibitory Factor; IGFBP-1: Insulin-like Growth Factor-Binding Protein; CXCR4: C-X-C Chemokine Receptor Type 4 CXCL12: C-X-C Motif Chemokine Ligand 12; MMP-2 and MMP-9: matrix metalloproteinase 2 and matrix metalloproteinase 9; uPA: Urokinase Plasminogen Activator; TIMPs: tissues inhibitors of metalloproteinases; COX-2: Cyclooxygenase-2; PGE2: Prostaglandin E2; eNOS: endothelial nitric oxide synthase; HLA-G, HLA-E, and HLA-C: human leukocyte antigens G, E, C; uNK Cells: Uterine Natural Killer Cells; HIF-1α: hypoxia-inducible factor 1-Alpha; Mcl-1: Myeloid cell leukemia 1; Bcl-xl: B-cell lymphoma-extra large; TRAIL/TRAIL-R: Tumor necrosis factor-Related Apoptosis-Inducing Ligand/receptor; TGF-β: transforming growth factor beta; Bcl-2: B-cell Lymphoma 2; FLIP: FLICE-Inhibitory Protein.

Placentation Step & Timeframe	Key Molecular Drivers & Mediators	Apoptotic & Cell Survival Markers	Functional Outcome & Critical Path
Step 1: Apposition & Adhesion (Days 6–7 post-fertilization)	L-Selectin (tethering interaction) αvβ3 & α4β1 Integrins (stable binding) E-cadherin (transient down-regulation) Lif (Leukemia Inhibitory Factor) IL-11 & MUC1 (epithelial receptivity)	Bcl-2/Bax Ratio: Heavily skewed toward survival (high Bcl-2) to maintain blastocyst structural integrity. Caspase-3: Undetectable/Inactive. Fas/FasL: Shielded to prevent maternal lymphocytic attack.	Definitive physical docking of blastocyst. Triggers transformation of uterine lining. Initiates focal matrix degradation for entry window.
Step 2: Decidualization (Days 8–12 post-fertilization)	Progesterone & cAMP (core triggers) dPR (Decidual Progesterone Receptor) IGFBP-1 & Prolactin (PRL) (phenotype markers) COX-2 & PGE2 (vascular permeability) Wnt4/β-catenin pathway	Surviving: Highly expressed in stromal cells to manage rapid cell shape change and growth. Bax: Temporarily elevated in non-receptive epithelium to allow focal shedding for embryo entry. p53: Kept low in stromal core.	Morphological switch of stromal cells to nutrient-rich, plump decidual cells. Builds a localized immunotolerant tissue bed. Forms a tight molecular boundary to check excessive digging.
Step 3: Trophoblast Proliferation & Early Invasion (Weeks 3–5 of gestation)	HIF-1α (Hypoxia-Inducible Factor 1-alpha) VEGF-A & VEGFR-1/Flt-1 Notch1 & Notch4 signaling Galectin-1 & PP13 HLA-G/HLA-E (non-classical MHC)	Mcl-1 & Bcl-xl: Strongly upregulated in the proliferating trophoblast core to block death paths. TRAIL/TRAIL-R: Selectively triggers shedding of maternal epithelial barriers while sparing fetal tissues. Caspase-8: Inhibited via FLIP.	Core creation of a vast cytotrophoblast shell. Syncytiotrophoblast expands to access maternal raw pools. Blocks early blood streaming via protective trophoblast plugs.
Step 4: Interstitial & Endovascular Invasion (Weeks 6–10 of gestation)	MMP-2 & MMP-9 (Gelatinases; matrix digestion) uPA (Urokinase Plasminogen Activator) Integrin Switch: α6β4 (epithelial) down-regulates; α1β1 & αvβ3 (vascular) up-regulate CXCR4 & CXCL12 chemokine axis ADAMTS proteases	Caspase-3/Caspase-9 Activation: Regulated and localized in old/fusing cytotrophoblasts to manage safe recycling into the syncytium. FasL (Fas Ligand): Highly expressed on EVTs to force invading maternal immune cells into apoptosis.	Deep digestion through extracellular tissue matrices into inner myometrium. Perfect physical mapping of EVTs alongside maternal spiral arteries. Establishes baseline physical network.
Step 5: Vascular Remodeling & Plug Dissolution (Weeks 11–14 of gestation)	PlGF (Placental Growth Factor) Angiopoietin-1 & Angiopoietin-2 eNOS (Endothelial Nitric Oxide Synthase) VE-cadherin (vascular mimicry integration) TGF-β & TIMPs (tissue inhibitors)	Endothelial Apoptosis: Maternal smooth muscle and inner lining cells are pushed into apoptosis by EVTs. Bcl-2/Bax Equilibrium: Stabilizes in villi to balance normal shedding into maternal pool. p53: Safeguards genetic copy accuracy.	Destroys arterial smooth muscle coat to make vessels broad and flaccid. Drops vascular resistance to feed a low-velocity, high-volume stream. Plugs dissolve safely, scaling oxygen supply.

## Data Availability

No new data were created or analyzed in this study. Data sharing is not applicable to this article.
